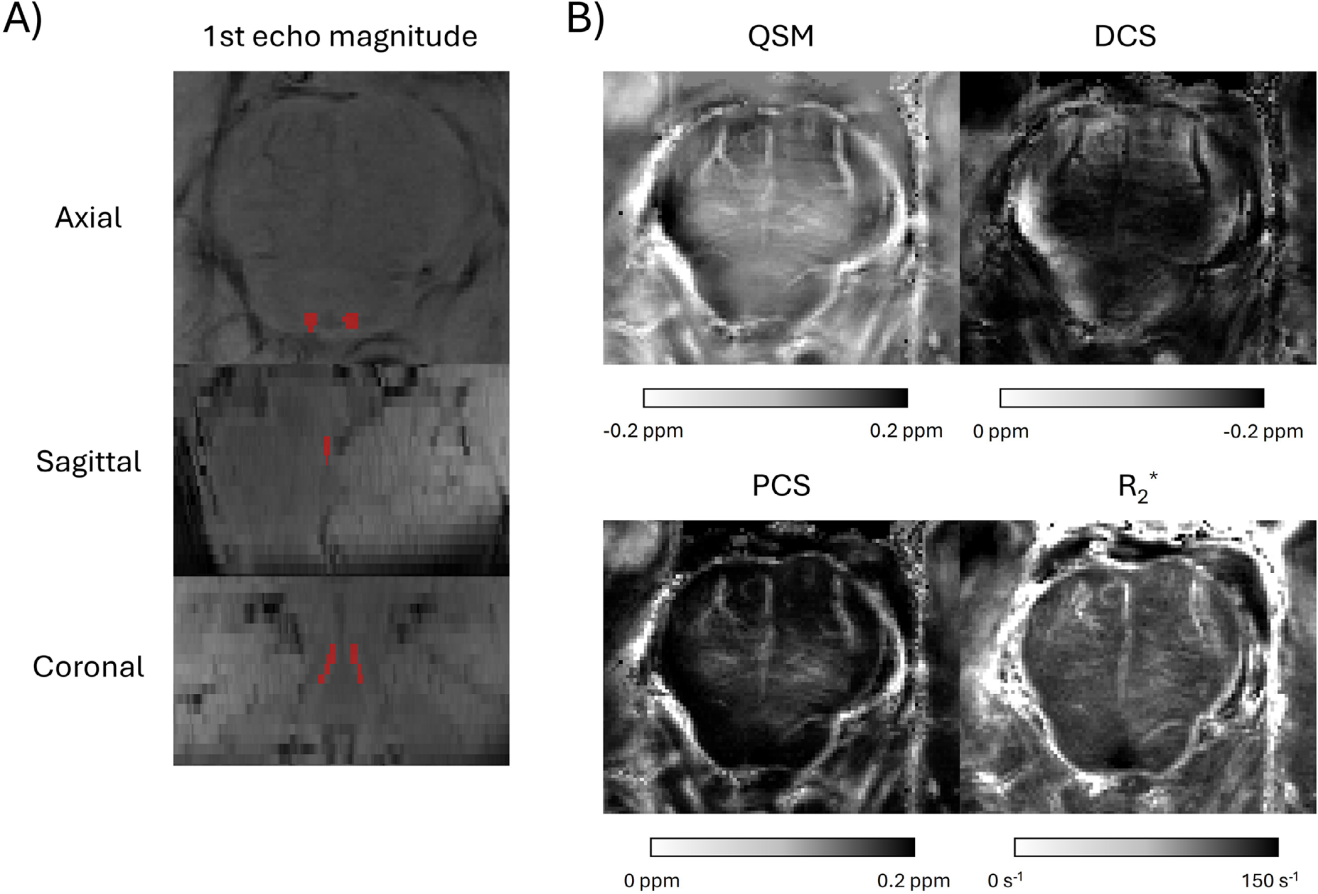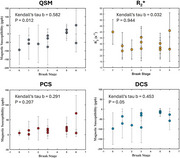# The magnetic susceptibility changes of Locus Coeruleus over tau pathology progression: a preliminary study

**DOI:** 10.1002/alz70856_106173

**Published:** 2026-01-08

**Authors:** Fabio Otsuka, Roberta Diehl Rodriguez, Chunlei Liu, Lea T. Grinberg, Maria Concepción Gracía Otaduy

**Affiliations:** ^1^ LIM44, Departamento de Radiologia e Oncologia, Faculdade de Medicina da Universidade de São Paulo, Sao Paulo, Brazil; ^2^ University of California, Berkeley, Berkeley, CA, USA; ^3^ Memory and Aging Center, UCSF Weill Institute for Neurosciences, University of California, San Francisco, San Francisco, CA, USA; ^4^ LIM44, Hospital das Clinicas HCFMUSP, Faculdade de Medicina, Universidade de Sao Paulo, Sao Paulo, Sao Paulo, Brazil

## Abstract

**Background:**

The magnetic susceptibility of Locus Coeruleus was evaluated against Braak staging in order to establish possible MRI biomarkers related to magnetic proeprties of LC during AD progression.

**Method:**

12 *postmortem* subjects were recruited. MRI as performed with a 7T scanner. Magnetic susceptibility (QSM), diamagnetic (DCS) and paramagnetic (PCS) components maps were calculated using the DECOMPOSE algorithm, with phase pre‐processing steps using the STISuite toolbox. The LC was manually segmented from the 1st echo magnitude images (Figure 1A) and mean values of each map was calculated on the entire segmented ROI. Alzheimer's pathological staging was performed according to Braak stage for AD pathology. To assess correlation between Braak staging and QSM, PCS and DCS the Kendall's tau test was used. Statistical significance was considered when *p* <0.05.

**Result:**

The lack of contrast on all images (Figure 1B) suggests that magnetic susceptibility effects are not the main factors for the LC contrast in MRI. However, the evaluation of these maps indicated that QSM continuously increases over Braak staging (Figure 2). This increase was found to be due to a decrease of DCS, rather than increase of PCS, which suggests that demyelination is the main contributing factor on the observed changes, rather than iron overload. The inclusion of more subjects is necessary to improve the statistical power of this study. The inclusion of other modalities, such as diffusion tensor imaging from MRI, spectroscopic measurements for metal concentration (mass spectrometry and electron paramagnetic resonance), and histopathological analysis for protein/metal burden may help better understand these alterations in LC over pathology progression.

**Conclusion:**

Our preliminary results suggest that LC undergoes magnetic changes mostly related to diamagnetic sources, potentially demyelination processes. No evidence of paramagnetic‐related effects were observed, indicating that iron overload effects are not sufficient to observe any changes. The inclusion of additional modalities may help improve these findings.